# Longitudinal Prediction of Divorce in Russia: The Role of Individual and Couple Drinking Patterns

**DOI:** 10.1093/alcalc/agt068

**Published:** 2013-07-12

**Authors:** Katherine Keenan, Michael G. Kenward, Emily Grundy, David A. Leon

**Affiliations:** 1 Faculty of Epidemiology and Population Health, London School of Hygiene and Tropical Medicine, Keppel Street, London WC1E 7HT, UK; 2 Department of Geography, University of Cambridge, Downing Place, Cambridge CB2 3EN, UK

## Abstract

**Aims:** The aim of the study was to explore associations between dimensions of alcohol use in married couples and subsequent divorce in Russia using longitudinal data. **Methods:** Follow-up data on 7157 married couples were extracted from 14 consecutive annual rounds (1994–2010) of the Russian Longitudinal Monitoring Survey, a national population-based panel study. Discrete-time hazard models were fitted to estimate the probability of divorce among married couples by drinking patterns reported in the previous survey wave. **Results:** In adjusted models, increased odds of divorce were associated with greater frequency of husband and wife drinking (test for trend *P* = 0.005, and *P* = 0.05, respectively), wife's binge drinking (*P* = 0.05) and husband's heavy vodka drinking (*P* = 0.005). Couples in whom the wife drank more frequently than the husband were more likely to divorce (OR 2.86, 95% CI 1.52–5.36), compared with other combinations of drinking. The association between drinking and divorce was stronger in regions outside Moscow or St. Petersburg. **Conclusion:** This study adds to the sparse literature on the topic and suggests that in Russia heavy and frequent drinking of both husbands and wives put couples at greater risk of future divorce, with some variation by region and aspect of alcohol use.

## INTRODUCTION

Alcohol use in Russia is a major public health concern (Leon *et al*., 2009), but little is known about the potential adverse effects of alcohol on drinkers' immediate families and households. This paper tries to fill the research gap by investigating the longitudinal relationship between alcohol and divorce in Russia.

### Alcohol in Russia

Together with a high annual per capita alcohol consumption (∼15–18 l (Leon *et al*., 2009)), the drinking pattern in Russia is particularly hazardous. A high proportion of total alcohol is drunk as spirits, up to 75% in some studies (Pomerleau *et al*., 2005; Popova *et al*., 2007), and research consistently finds that over half of men are binge drinkers (Nilssen *et al*., 2005; Perlman, 2010). Alcohol use is normalized and incorporated into everyday life, and there is high tolerance for heavy episodic drinking with the intention of intoxication (Saburova *et al*., 2011). Heavy drinking is more common in youth and middle age, among those with low education, the unemployed and those in poorer households (Bobak *et al*., 1999; Tomkins *et al*., 2007; Perlman, 2010; Cook *et al*., 2011). However, the biggest disparity is by gender: men drink more frequently, more as spirits and are more likely to binge drink (Bobak *et al*., 1999; Malyutina *et al*., 2001; Bobrova *et al*., 2010). Heavy drinking is considered more socially acceptable for men and is perceived to play an important role in male social life, professional life and stress management (Mustonen, 1997; Pietilä and Rytkönen, 2008a).

### Divorce in Russia

Russia has long had one of the highest divorce rates in Europe. Between 1960 and 1995, the crude divorce rate quadrupled (Avdeev and Monnier, 2000) and has fluctuated considerably since the 1990s. The fact that obtaining a divorce is quick, simple and free (Antokolskaia, 2002) may partly explain the high divorce rate, but micro-level factors are also important. In Russia, divorce is associated with young age, length of union, frequent migration, childlessness, experience of parental divorce and premarital conception (Jasilioniene, 2007; Muszynska and Kulu, 2008), but few studies consider alcohol as a potential risk factor, despite the high prevalence of hazardous drinking.

### Divorce and Alcohol

Outside of Russia, many cross-sectional studies find an association between drinking and divorce, both at individual (Hasin *et al*., 2007; Joutsenniemi *et al*., 2007) and aggregate levels (Caces *et al*., 1999). However, in order to understand the direction of causation, longitudinal data are necessary. A small number of longitudinal studies have found that heavy drinking predicts subsequent divorce (Leonard and Rothbard, 1999; Collins *et al*., 2007), and increased marital dysfunction (Marshal, 2003).

The contribution of alcohol to family disruption in Russia has been discussed, but only a few studies presented interpretable data on the topic (Stack and Bankowski, 1994; Carlson and Vagero, 1998; Taitz, 2005; Osadchiya *et al*., 2008). Since the Soviet period alcoholism has been a commonly cited reason for divorce (White, 1996; Osadchiya *et al*., 2008). Drinking is perceived to be a leading cause of couple conflict (Vannoy *et al*., 1999; Pishnyak, 2009) and increases the risk of domestic violence (Cubbins and Vannoy, 2005; Lisova, 2008; Stickley *et al*., 2008; Zhan *et al*., 2011). The only study we have found that investigated drinking and divorce, specifically (Stack and Bankowski, 1994), was a cross-sectional analysis, similar to other studies (Taitz, 2005; Tomkins *et al*., 2007). To date, no longitudinal studies of the topic have been conducted in Russia.

In previous studies, discordant couples, where one person drinks more than the other, have been found to have the highest risk of dissatisfaction, disruption and conflict (Ostermann *et al*., 2005; Homish and Leonard, 2007; Meiklejohn *et al*., 2012). It is not known whether the same pattern operates in Russia where couple drinking pattern discordance is the norm, and is commonly attributed to cultural notions of masculinity and femininity (Pietilä and Rytkönen, 2008b). Drinking is considered part of the male breadwinner role, something that ‘real men’ do. By contrast, wives' household and childrearing responsibilities prevent heavy drinking, and women who do so may be negatively perceived (Bobrova *et al*., 2010). Wives may help to maintain their husband's masculine role by providing alcohol with meals (Pietilä and Rytkönen, 2008b), giving them money for alcohol and bringing alcohol to them if they are sick or disabled (Saburova *et al*., 2011). It is common for wives to try to informally control their husband's drinking, but this is also a source of conflict (Holmila, 1987). Such acceptance of heavy drinking on the part of wives may mediate any relationship between drinking and divorce, creating a weaker association in Russia than in other populations.

## AIMS

In this paper, we examine longitudinal associations between drinking patterns and subsequent risk of divorce in a sample of Russian couples. Firstly, we explore how variations in individual spouse drinking frequency and volume are associated with probability of subsequent divorce; secondly, whether spousal drinking discordance is associated with subsequent divorce.

## DATA AND METHODS

### Data

The analysis uses the Russian Longitudinal Monitoring Survey (RLMS) (Higher School of Economics *et al*., 1992–present), a Russian household panel survey started in the early 1990s to monitor the effect of political transition on health and well-being. The study was primarily designed as a repeated cross-sectional survey based on dwelling but the design permits longitudinal analysis. We used the data from phase 2 (1994–2010, waves 5–19).

Full details on RLMS design and sampling are available on the website (http://www.cpc.unc.edu/projects/rlms-hse). At the beginning of phase 2 (1994), a three-stage probability sample was chosen. The final sample consisted of 4718 dwellings, of which 84.3% completed interviews (lower in the Moscow/St. Petersburg regions (60.2%)). According to the RLMS survey team, the population sampled in wave 5 (1994) compared well with the 1989 census population in terms of distribution of household size, sex, age and urban-rural residence. Divorce rates in the RLMS were slightly lower than national rates, but followed the same pattern over time.

The units of analysis were married couples, identified as such through the household roster, but only included if both reported being married and both had completed individual interviews. On this basis, ∼20% of couples were excluded. Both spouses were linked to their follow-up data from the next wave. If follow-up data for either party were missing, their spouses' data about their marital status were used to ascertain the outcome.

## VARIABLES

### Outcome: divorce

An event (divorce) was defined if *both* parties reported their marital status as divorced. There were 94 cases of spousal disagreement about divorce, and sensitivity analysis was carried out where disagreeing couples were included in the event group.

### Main exposure: alcohol consumption of both spouses

At each wave, participants were asked about drinking frequency, beverage types they consumed and the maximum daily volume of each beverage consumed in the 30 days before interview. The drinking information collected in each wave was used as the exposure variable for the follow-up period ending at the next wave.

Frequency of drinking was categorized into groups: abstained/2–3 times a month/weekly/2–3 times a week/4+ times a week. We also derived a ‘drinking pattern’ variable which classified individuals into binge drinkers, non-binge drinkers or abstainers. Binge drinking was defined as consuming >80 g of ethanol from a single type of beverage on a single occasion, a cut-off used in previous studies in Russia (Malyutina *et al*., 2001; Bobak *et al*., 2004). The third variable was the usual amount of ethanol consumed from spirits in a single episode, divided into fifths for simplicity. Spirit consumption has been previously used in Russia as an indicator of heavy drinking (Bobak *et al*., 1999; Pomerleau *et al*., 2008). This was done for men only, as female spirit consumption was very low. Respondents were asked how many grams of ‘vodka or other hard liquor’ they usually consumed in a day, and this was converted into grams of ethanol, assuming an ethanol concentration of 0.43 or 43% by volume. The distribution of grams of spirits was skewed to the right, and the range in the 5th percentile was 10–100 g and in the 95th percentile was 550–3000 g. A minority (1%) reported usually drinking over 1000 g a day; we excluded respondents who said they drank >4000 g in a day on the basis of implausibility (2 cases). We also considered beer and wine consumption using the same method.

Two constructed categorical variables represented spousal drinking concordance. Drinking frequency concordance had four groups: neither frequent drinkers (where frequent drinking was at least twice a week), wife frequent drinker and husband not; husband frequent drinker and wife not or both frequent drinkers. The same was done for drinking pattern concordance but with binge drinking instead of frequent drinking.

### Other variables

From previous studies (Lyngstad and Jalovaara, 2010), several factors were identified as potential confounders. However, many of these, such as employment, could be on the causal pathway. We included these in the model but took care in interpreting the effects of adjustment.

All covariate data were self-reported and taken from the start of each interval. We included individual level variables for both spouses (age, education, employment, life satisfaction, economic security and health status) and couple-level variables (shared biological children and household income). A binary variable identified whether the couple had resident young (<18 years) biological children. Exact age was divided into 10-year groups; and education had three categories: incomplete secondary; secondary, specialist and professional; and university level and above. Employment status had three categories: unemployed; employed and ‘other’—including students, housewives, etc. Household income was adjusted for equivalized household size, using the OECD-modified scale (Hagenaars *et al*., 1996), and then divided into deciles. Life satisfaction was reported on a 5-point scale, and dichotomized into poor life satisfaction (not at all satisfied/less than satisfied) versus rather satisfied/very satisfied. Subjective economic insecurity was measured by the question ‘How concerned are you that you might not be able to provide yourself with the bare essentials in the next 12 months?’, and the 5-point scale classified into two groups of very concerned (the most negative category) versus the rest. Respondents' self-assessed health was reported on a 5-point scale and grouped into the two worst categories (very poor/poor) versus the rest (average/good/very good). Variables for life satisfaction, economic insecurity and health were dichotomized due to small numbers. For geography, a categorical variable classified areas of Russia into four regions: Central, Urals, North & Northwest/metropolitan areas (Moscow and St. Petersburg)/Volga and the North Caucasus/Siberia and the Far East.

### Analytical approach

The data were modelled using a discrete-time hazard model (Fahrmeir, 1998) in which the probability of an event, here divorce, between successive time points *t*-1 and *t* is expressed conditionally on not being divorced, and on the values taken by other relevant covariates, at time point *t*-1. Because of the conditional structure of the model, the log likelihood components from each time point are independent and so can be summed to allow a single overall fit using standard logistic regression. This approach is described as ‘pooled logistic regression’ in epidemiological and demographic studies (D'Agostino *et al*., 1990; Grundy and Kravdal, 2008). The analysis assumes that the divorce took place between time point *t*-1 and *t* and that the coefficients are non-time-varying*.* All analysis was performed in STATA 12 (StataCorp, 2011).

To assess the hypothesis of a relationship between spouse drinking discordance and subsequent divorce, we first did a formal interaction test using likelihood ratio tests, then fitted separate models using the constructed couple-level alcohol variables, adjusted for individual drinking.

We accounted for the multi-stage sampling design by calculating robust standard errors, adjusted for clustering by primary sampling unit. We explored the effect of missing data by fitting multiple imputation models under the missing-at-random (MAR) assumption (Carpenter and Kenward, 2013), and then compared the results with a complete case analysis.

## RESULTS

### Descriptive statistics

The follow-up rate of couples between successive waves ranged from 83 to 93% (Table 1). Couple drop-out was associated with higher education and younger age, but not alcohol use. The sample with follow-up data consisted of 7157 individual couples, contributing 30,900 couple follow-up periods. Of these, 1950 (6%) had outcome data from just one party.

**Table 1.** Number of follow-up observations for each interval

Interval by wave number	Calendar year	Column 1	Column 2	Column 3
		Married couples at the start of each follow-up period (*n*)	Couples completely lost to follow-up by next wave [*n* (% of column 1)]	Couples with follow-up and outcome data [*n* (% of column 1)]
5–6	1994–1995	2690	455 (16.9)	2234 (83.1)
6–7	1995–1996	2392	297 (12.4)	2095 (87.6)
7–8	1996–1998	2340	340 (14.5)	1998 (85.4)
8–9	1998–1999	2193	247 (11.3)	1946 (88.7)
9–10	1999–2000	2194	193 (8.8)	2001 (91.2)
10–11	2000–2001	2325	169 (7.3)	2156 (92.7)
11–12	2002–2003	2389	191 (8.0)	2198 (92.0)
12–13	2003–2004	2365	218 (9.2)	2147 (90.8)
13–14	2004–2005	2345	218 (9.3)	2126 (90.7)
14–15	2005–2006	2279	215 (9.4)	2064 (90.6)
15–16	2006–2007	2719	274 (10.1)	2444 (89.9)
16–17	2007–2008	2715	282 (10.4)	2432 (89.6)
17–18	2008–2009	2637	201 (7.6)	2435 (92.3)
18–19	2009–2010	2820	196 (7.0)	2624 (93.1)
Total		34,403	3496 (10.2)	30,900 (89.8)

Over the 15 time intervals, 344 divorces were observed. Couples who divorced were significantly younger and more likely to live in Moscow or St. Petersburg and to be childless. Individual factors associated with divorce were either party drinking at least twice a week or having poor life satisfaction, wife's binge drinking and wife's poor self-assessed health. No associations were seen with education, employment or economic security. Divorce was lowest when both were non-frequent drinkers or non-binge drinkers. Husbands drank more frequently, were less likely to abstain and were more likely to binge drink than wives (tabulations not shown). Almost half (47%) of husbands were binge drinkers compared with only 10% of wives. As has been found in previous research (Meiklejohn *et al*., 2012), the most common couple drinking pattern was concordance where both parties were non-frequent or non-binge drinkers.

### Multivariable analysis

Table 2 presents a summary of five models with adjusted odds ratios for divorce according to husband's/wife's drinking frequency, husband's usual amount of vodka per occasion and husband's/wife's drinking pattern. Covariates were added to the models in four groups: Model 1 was adjusted for age, calendar time and education; model 2 was additionally adjusted for shared children, life satisfaction and health; model 3 added employment, household income and economic security and model 4 included individual spouse drinking.

**Table 2.** Adjusted odds of divorce according to husband's and wife's drinking frequency and pattern

	Model 1: age, calendar time and education [OR (95% CI)]	Model 2: plus shared children, health and life satisfaction [OR (95% CI)]	Model 3: plus socio-economic factors [OR (95% CI)]	Model 4: plus spouse's drinking [OR (95% CI)]
**Husband**				
Drinking frequency (*n* = 29,403)				
Abstainer	1.09 (0.81–1.45)	1.07 (0.80–1.44)	1.09 (0.82–1.45)	1.06 (0.78–1.43)
1–3 times a month	1.00 (ref)	1.00 (ref)	1.00 (ref)	1.00 (ref)
Once/week	1.42* (1.06–1.91)	1.43* (1.07–1.92)	1.43* (1.07–1.91)	1.38* (1.02–1.87)
2–3 times/week	1.93** (1.35–2.74)	1.92** (1.34–2.74)	1.90** (1.32–2.72)	1.73** (1.23–2.45)
4+ times/week	1.98* (1.16–3.38)	1.95* (1.16–3.30)	1.87* (1.08–3.23)	1.64 (0.92–2.90)
Test for trend among drinkers	*P* = 0.001	*P* = 0.001	*P* = 0.002	*P* = 0.005
Drinking pattern (*n* = 29,569)				
Abstainer	0.88 (0.62–1.25)	0.86 (0.60–1.23)	0.88 (0.62–1.25)	0.87 (0.62–1.22)
Non-binge drinker	1.00 (ref)	1.00 (ref)	1.00 (ref)	1.00 (ref)
Binge drinker	1.16 (0.92–1.45)	1.14 (0.91–1.43)	1.14 (0.91–1.43)	1.10 (0.88–1.38)
Usual amount of vodka on a single occasion (fifths) (*n* = 15,703)				
1 (lowest)	1.00 (ref)	1.00 (ref)	1.00 (ref)	–
2	0.90 (0.59–1.36)	0.90 (0.59–1.36)	0.90 (0.59–1.36)	–
3	1.16 (0.83–1.61)	1.15 (0.83–1.59)	1.14 (0.82–1.59)	–
4	1.32 (0.91–1.93)	1.31 (0.90–1.89)	1.30 (0.89–1.89)	–
5 (highest)	1.99** (1.32–3.01)	1.96** (1.29–2.96)	1.91** (1.25–2.93)	–
Test for trend	*P* = 0.005	*P* = 0.005	*P* = 0.008	–
**Wife**				
Drinking frequency (*n* = 29,490)				
Abstainer	0.98 (0.76–1.26)	0.98 (0.76–1.26)	1.03 (0.80–1.33)	1.06 (0.81–1.38)
1–3 times a month	1.00 (ref)	1.00 (ref)	1.00 (ref)	1.00 (ref)
Once/week	1.32 (0.95–1.83)	1.35 (0.97–1.87)	1.33 (0.95–1.85)	1.16 (0.83–1.63)
2+ times/week	2.08* (1.18–3.65)	2.08* (1.19–3.64)	2.09** (1.19–3.66)	1.65 (0.97–2.81)
Test for trend among drinkers	*P* = 0.027	*P* = 0.023	*P* = 0.053	*P* = 0.251
Drinking pattern (*n* = 29,569)				
Abstainer	0.95 (0.73–1.24)	0.93 (0.71–1.22)	1.00 (0.75–1.32)	1.00 (0.75–1.32)
Non-binge drinker	1.00 (ref)	1.00 (ref)	1.00 (ref)	1.00 (ref)
Binge drinker	1.45** (1.12–1.87)	1.43** (1.10–1.87)	1.41* (1.08–1.84)	1.41* (1.08–1.84)

*P < 0.05.

**P < 0.1.

***P < 0.001.

After adjustment, there was a significant positive trend between husband's drinking frequency, husband's usual amount of vodka and divorce. When husbands drank 2–3 times per week, compared with 1–3 times a month, couples had 73% higher odds of divorce. The association attenuated only slightly on addition of socio-economic factors and spouse drinking. Where the husband was a heavy spirit drinker (the top fifth of vodka consumption), the adjusted odds of divorce at follow-up were twice as high as those of a light spirit drinker. Couples in whom the wife drank frequently, or was a binge drinker, were associated with higher odds of divorce. After adjustment for husband's drinking, the association with frequency attenuated, but the odds for binge drinking remained significant. The positive trend between divorce and spirit consumption was not found when beer and wine consumption on a single occasion were considered, but there was some evidence that women drinking over 20 g of ethanol as beer had an increased risk of divorce (results not shown). We experimented with using gender-specific cut-offs to identify binge drinking (men ≥80 g ethanol, women ≥60 g ethanol), but this made little difference to the results. The effect of male drinking frequency varied significantly by region (*P* = 0.002), having a very strong association in Volga and the North Caucasus, but weaker or reverse effects in Moscow or St. Petersburg (Table 3).

**Table 3.** Adjusted odds of divorce according to husband's drinking frequency and area in Russia

Formal test for interaction by area (*P* = 0.006)	Area 1: Central, Ural, North and Northwest [OR (95% CI)]	Area 2: Moscow and St. Petersburg [OR (95% CI)]	Area 3: Volga and North Caucasus [OR (95% CI)]	Area 4: Siberia and Far East [OR (95% CI)]
Husband's drinking frequency^a^				
Abstainer	0.86 (0.49–1.53)	0.90 (0.53–1.50)	1.51* (1.01–2.25)	0.83 (0.47–1.46)
1–3 times a month	1.00 (ref)	1.00 (ref)	1.00 (ref)	1.00 (ref)
Once/week	1.49 (0.96–2.31)	1.08 (0.38–3.14)	1.44 (0.70–2.95)	1.61 (0.75–3.46)
2–3 times/week	2.48*** (1.50–4.09)	0.32 (0.05–2.16)	1.85** (1.16–2.96)	1.95 (0.97–3.93)
4+ times/week	1.27 (0.56–2.87)	0.34*** (0.26–0.44)	4.42*** (2.22–8.81)	1.30 (0.32–5.38)
*n* (observations)	11,717	2392	9974	5320

^a^Adjusted for age, education, wave, life satisfaction, shared children, health and socio-economic factors.

*P < 0.05.

**P < 0.01.

***P < 0.001.

Although the interaction between husband's and wife's drinking was non-significant (*P* = 0.19), regressions using the constructed variable showed that couples in whom the wife drank more frequently had higher odds of divorce than any other category.

The results of a sensitivity analysis using an alternative way of determining divorce (including couples who disagreed about being divorced as cases in the numerator) showed no substantial differences. We show results from a complete case analysis as the analysis using multiple imputation showed the same pattern of association.

## DISCUSSION

We have found that in a large population-sample of married couples in Russia, regular drinking and binge drinking by either spouse were independently associated with an increased risk of subsequent divorce, relative to couples who drank moderately. There was a dose–response effect with the risk of divorce increasing with frequency and usual volume of spirits drunk by the husband on a single occasion. This effect was not accounted for by age, life satisfaction, children, health or socio-economic factors. Discordant spousal drinking patterns were associated with higher odds of divorce only when the wife drank more frequently. This may relate to the norms of Russian drinking culture where heavy drinking is considered socially acceptable for men, but not for women (Bobrova *et al*., 2010). On the whole, the results suggest that both high frequency and large volumes of alcohol are significant threats to marital stability in Russia, as has been found in UK and US populations, but there are variations according to features of alcohol use and region.

The geographical variation in the level of effect could be explained by factors such as religion, which was not included because only available for waves 9–12. This may explain the stronger association in Volga and North Caucasus, the region containing the highest proportions of both Muslims and abstainers (20.1% were Muslims, compared with 7.2% in Moscow & St. Petersburg). However, small numbers prohibited further exploration. The variation is unlikely to be explained by differential response rates between regions, because results using multiple imputation showed the same pattern of association.

There were some limitations related to the survey questionnaire. Firstly, alcohol was measured by self-report, where under-reporting is likely. However, provided that under-reporting remains constant over time, this should not bias our associations. Secondly, binge drinking may be underestimated as we could not capture combinations of beverages drunk at each occasion. Lastly, we could not explore the effects of alcohol on cohabiting partnerships because that data were not collected at every wave.

Panel studies like the RLMS are likely to suffer from selection bias because heavy drinkers and those with family problems are both less likely to take part and more likely to drop out (Torvik *et al*., 2011). As divorce generally declines with age, and drop-out was higher in younger couples, we may have used a sample of disproportionately stable couples. However, the RLMS divorce rates compared well with national rates (data not shown).

The statistical modelling assumed that the relationship between drinking and divorce remained stable over time (1994–2010). We tested this by adjusting the models for calendar time and formally tested for interactions, finding no evidence.

This study adds to the literature by using longitudinal data, rather than cross-sectional. Although the temporal separation of drinking and divorce rules out reverse causality, we are not claiming that the results definitively demonstrate that alcohol causes divorce. The possibility remains of residual confounding by unmeasured factors, like conflict, mental illness, personality characteristics, parental divorce, union duration or relationship history. Further research could explore the potential pathways for how alcohol might lead to divorce, perhaps using methods such as structural equation modelling.

## CONCLUSIONS

This study finds that heavy and frequent drinking by either spouse increases the risk of subsequent divorce in Russia, and the association varies geographically and by aspects of alcohol use. This provides longitudinal evidence that alcohol is not just associated with individual harms, but with harm to family relationships. Given that Russia faces a serious alcohol problem, this finding supports the case for stricter control to prevent not only adverse health problems but also adverse social and family outcomes.

## Funding

At the time of the research, K.K. was funded by an Economic and Social Research Council PhD studentship. Funding to pay the Open Access publication charges for this article was provided by Research Councils UK (RCUK).

*Conflict of interest statement.* None declared.
